# Psychiatric disorders in female psychosexual disorders—a nationwide, cohort study in Taiwan: Psychiatric disorders and female psychosexual disorders

**DOI:** 10.1186/s12888-021-03060-1

**Published:** 2021-01-28

**Authors:** Iau-Jin Lin, Nian-Sheng Tzeng, Chi-Hsiang Chung, Wu-Chien Chien

**Affiliations:** 1 Graduate Institute of Life Sciences, National Defense Medical Center, 9314R, No.161, Section 6, Min-Chuan East Road, Neihu District, Taipei, 11490 Taiwan, Republic of China; 2 Department of Psychiatry, School of Medicine, Tri-Service General Hospital, National Defense Medical Center, Taipei, Taiwan, Republic of China; 3 Student Counseling Center, National Defense Medical Center, Taipei, Taiwan, Republic of China; 4 Department of Medical Research, Tri-Service General Hospital, National Defense Medical Center, 7115R, No.325, Section 2, Cheng-Gung Road, Neihu District, Taipei, 11490 Taiwan, Republic of China; 5 School of Public Health, National Defense Medical Center, Taipei, Taiwan, Republic of China; 6 Taiwanese Injury Prevention and Safety Promotion Association, Taipei, Taiwan, Republic of China

**Keywords:** Psychosexual disorders, Affective disorders, Females, National Health Insurance Research Database, Cohort study

## Abstract

We aimed to investigate whether females with psychosexual disorders were associated with the risk of affective and other psychiatric disorders. A total of 2240 enrolled individuals, with 560 patients with psychosexual disorders and 1680 subjects without psychosexual disorders (1:3) matched for age and index year, from the Longitudinal Health Insurance Database, retrieved from the National Health Insurance Research Database (NHIRD), between 2000 and 2015 in Taiwan. The multivariate Cox regression model was used to compare the risk of developing psychiatric disorders during the 15 years of follow-up. There were 98 in the cohort with psychosexual disorders (736.07 per 100,000 person-year) and 119 in the non-cohort without psychosexual disorders (736.07 per 100,000 person-year) that developed psychiatric disorders. The multivariate Cox regression model revealed that the adjusted hazard ratio (HR) was 9.848 (95% CI = 7.298 — 13.291, *p* < 0.001), after the adjustment of age, monthly income, urbanization level, geographic region, and comorbidities. Female patients with psychosexual disorders were associated with the risk of psychiatric disorders. This finding could be a reminder for clinicians about the mental health problems in patients with psychosexual disorders.

**Supplementary Information:**

The online version contains supplementary material available at 10.1186/s12888-021-03060-1.

## Background

Psychosexual disorders could be classified into sexual dysfunctions, paraphilias, and gender identity disorders [1, 2], and these psychosexual disorders are regarded as part of the psychiatric disorders [3]. Previous studies have shown that female patients with psychosexual disorders, such as sexual dysfunctions, paraphilias, and gender identity disorders, would suffer from emotional distress, social embarrassment, and even stigmatization [4, 5].

Several researchers have shown the neurodevelopmental interlinks between the psychosexual and psychiatric disorders: Sex differences in the microglial function might partially explain the differences observed in susceptibilities and outcomes of the neuropsychiatric disorders in men and women [6]. Rajkumar (2014) pointed out that both gender identity disorders and schizophrenia are associated with altered cerebral sexual dimorphism and changes in cerebral lateralization [7]. Previous studies have also found that endocrine factors are related to female psychosexual disorders. For example, sex steroids, such as estrogen or progestin, insufficiency may adversely affect central sexual thought processes, and contribute to the female sexual dysfunctions, such as hypoactive sexual desire disorder [8]. Also, gender dysphoria may have several genes involved in the sex hormone–signaling in the brains [9]. Sex hormones such as estrogen have many effects on anxiety and depression [10]. Several studies have found mutual relations between psychiatric comorbidity and psychosexual disorders [11–16]. For the clinicians, it is essential to better understand the mutual relationship between female patients with psychosexual disorders and their psychiatric morbidity. And these psychiatric disorders might well contribute to the distress, disability, or an increased risk of suffering death, pain, or disability, and consequent behavioral, psychological, or biological dysfunctions [3, 17]. Therefore, several neurodevelopmental, endocrine and psychological factors could be the linkage between psychosexual and psychiatric disorders.

Previous studies have found that depressive disorders are frequently associated with sexual dysfunction, across all the phases of sexual responses [18], and the attention problems related to anxiety might impair sexual motivation even with adequate stimuli [19]. Besides, sexual dysfunction is frequent in patients with posttraumatic stress disorder [20, 21]. However, some researchers have revealed that no psychiatric comorbidity was found in female patients with gender identity disorder [22, 23]. Furthermore, the relationship between female paraphilia and psychiatric disorders remains unclear, since patients with female paraphilia are rare [24, 25]. Therefore, depression, anxiety, and trauma-related disorders are associated with sexual dysfunctions, and also with the association between psychiatric disorders and paraphilia and gender identity disorder. Besides, there is a gap in the literature that no previous cohort studies have been conducted to examine the risk of psychiatric disorders in female patients with psychosexual disorders. We hypothesize that these psychosexual disorders are associated with the risk of psychiatric disorders in a long-term follow-up. We, therefore, conduct the present study, using Taiwan’s National Health Insurance Research Database (NHIRD), to investigate the association between psychosexual disorders and psychiatric disorders, in a 15-year follow-up.

## Methods

### Data sources

The National Health Insurance (NHI) Program was launched in Taiwan in 1995, and as of June 2009, including contracts with 97% of the medical providers, with approximately 23 million beneficiaries, or more than 99% of the entire population [26]. The National Health Insurance Research Database (NHIRD) uses the International Classification of Diseases, 9th Revision, Clinical Modification (ICD-9-CM) codes to record the diagnoses [27]. The present study has used the NHIRD to identify the inpatients with a discharge diagnosis of psychosexual disorders based on the ICD-9-CM codes, including sexual dysfunctions, paraphilia, and gender identity disorders, during 2000–2015. The paraphilias included the diagnoses as exhibitionism, fetishism, frotteurism, pedophilia, sexual masochism, sexual sadism, transvestic fetishism, voyeurism, other paraphilia, and paraphilia, not otherwise specified [3]. All the ICD-9-CM codes of psychosexual disorders are as listed in Table S1. In this database, all the personal identification data were enciphered, for the protection of the privacy of the patients. The records of ambulatory care visits and inpatient claims periodically were reviewed randomly by the NHI Administration to verify the accuracy of the diagnoses [28]. Several previous studies have documented the details of the program [29–33].

### Study design and sampled participants

Patients with newly diagnosed psychosexual disorders were selected from the 2 million Longitudinal Health Insurance Database (LHID), randomized retrieved from the NHIRD, which covers 99% of the entire population of Taiwan, between January 1, 2000, and December 31, 2015. The patients with psychosexual disorders before 2000 were excluded. Besides, the patients diagnosed with psychiatric disorders before 2000, or before their first visit for any psychosexual disorder, were also excluded. In Taiwan, the legal age of full civil competency is 20 years of age, according to Taiwan’s Civil Code [34], therefore, all patients aged < 20, were excluded as well. In this study, 560 patients with the psychosexual disorder and 1680 subjects without psychosexual disorders, were 1:3 matched, for age and index-year control, with a statistic power of 0.72 [35], and little power improvement resulted from increasing the number of controls while the ratio beyond 1:3 or 1:4 [36]. Therefore, the present study is a population-based, matched cohort study.

### Covariates

The covariates included age groups (20–49, ≥ 50 years), geographical area of residence (north, center, south, and east of Taiwan), urbanization level of residence (levels 1 to 4), and monthly income (in New Taiwan Dollars [NT$]; < 18,000, 18,000-34,999, ≥35,000). The urbanization level of residence was defined according to the population and various indicators of the level of development. Level 1 was defined as a population of > 1,250,000, and a specific designation as political, economic, cultural, and metropolitan development. Level 2 was defined as a population between 500,000 and 1,249,999, and as playing an important role in the politics, economy, and culture. Urbanization levels 3 and 4 were defined as a population between 149,999 and 499,999, and < 149,999, respectively.

### Comorbidity

We assessed the comorbidities by using the Charlson Comorbidity Index (CCI), which categorizes comorbidities using the ICD-9-CM codes, and scores each comorbidity category [37–39]. The CCI is used for comorbidity adjustment as a useful measure and substitutes for the usage of the individual comorbidity variables in health services research [40]. In CCI, the comorbidities include myocardial infarction, congestive heart failure, peripheral vascular disease, cerebrovascular disease, chronic obstructive pulmonary disease, dementia, paralysis, diabetes mellitus, diabetes with sequelae, chronic renal failure, cirrhosis of the liver, moderate-severe liver disease, peptic ulcers, rheumatoid arthritis, and AIDS [41]. The combination of all the scores was regarded as a single comorbidity score. A score of zero indicates that no comorbidities were found, and higher scores indicate higher comorbidity burdens [42].

### Outcome measures

Enrolled individuals in these two cohorts were tracked for 15 years, starting from the index date, to identify those who developed psychiatric disorders, comprising dementia, anxiety disorders, depressive disorders, bipolar disorders, eating disorders, sleep disorders, and psychotic disorders, withdrew from the NHI program, or reached the end of 2015. All the ICD-9-CM codes of psychiatric disorders are as listed in Table S1.

### Statistical analysis

All statistical analyses were performed using the SPSS for Windows, version 22.0 (IBM Corp., Armonk, NY). χ^2^ and t-tests were used to appraise the distributions of the categorical and continuous variables, respectively. The multivariate regression model was used to determine the risk of psychiatric disorders since death can act as a competing risk factor for psychiatric disorders [43, 44]. The results were presented as a hazard ratio (HR) with a 95% confidence interval (CI). Differences in the risk of psychiatric disorders between the study and control groups were estimated using the Kaplan-Meier method with the log-rank test. A 2-tailed *p-*value < 0.001 was considered to indicate a statistical significance, to minimize the type I error as possible.

## Results

### Sample characteristics

There was no significant difference between these two cohorts in age, marital status, education, insured monthly premiums, and the CCI scores. The cohort with psychosexual disorders tended to search for medical help in summer, autumn, and winter. Furthermore, the cohort with psychosexual disorders tended to live in the north, and the offshore islands resided more in the region of urbanization level 2 and received their medical treatments in the medical centers (Table 1).

**Table 1:** Characteristics of study at the baseline

Psychosexual disorders	Total		With		Without		***P***
Variables	n	%	n	%	n	%	
**Total**	2240		560	25.00	1680	75.00	
**Age (years)**	35.08 ± 12.99		34.70 ± 11.46		35.21 ± 13.46		0.421
**Age group (years)**							0.999
20–49	2016	90.00	504	90.00	1512	90.00	
≧50	224	10.00	56	10.00	168	10.00	
**Married**							0.692
Yes	936	41.79	230	41.07	706	42.02	
No	1304	58.21	330	58.93	974	57.98	
**Education (years)**							0.823
< 12	568	25.36	144	25.71	424	25.24	
≧12	1672	74.64	416	74.29	1256	74.76	
**Insured premium (NT$)**							0.663
< 18,000	1974	88.13	499	89.11	1475	87.80	
18,000-34,999	184	8.21	41	7.32	143	8.51	
≧35,000	82	3.66	20	3.57	62	3.69	
**CCI_R**	0.39 ± 1.37		0.30 ± 0.94		0.42 ± 1.48		0.081
**Season**							< 0.001
Spring (March–May)	581	25.94	91	16.25	490	29.17	
Summer (June–August)	602	26.88	154	27.50	448	26.67	
Autumn (September–November)	476	21.25	147	26.25	329	19.58	
Winter (December-Februrary)	581	25.94	168	30.00	413	24.58	
**Location**							< 0.001
Northern Taiwan	945	42.19	308	55.00	637	37.92	
Middle Taiwan	581	25.94	112	20.00	469	27.92	
Southern Taiwan	574	25.63	112	20.00	462	27.50	
Eastern Taiwan	126	5.63	21	3.75	105	6.25	
Outlets islands	14	0.63	7	1.25	7	0.42	
**Urbanization level**							< 0.001
1 (The highest)	784	35.00	126	22.50	658	39.17	
2	1029	45.94	357	63.75	672	40.00	
3	112	5.00	21	3.75	91	5.42	
4 (The lowest)	315	14.06	56	10.00	259	15.42	
**Level of care**							< 0.001
Medical center	798	35.63	357	63.75	441	26.25	
Regional hospital	595	26.56	147	26.25	448	26.67	
Local hospital	847	37.81	56	10.00	791	47.08	

*P*: Chi-square / Fisher exact test on category variables and t-test on continue variables

Without married: un-married, divorce, spouse death, and unknown

Education years < 12: elementary school, junior high school, (vocational) high school, and unknown; Education years ≧12: university, college, and graduate

*CCI_R* Charlson comorbidity index removed dementia

### The cumulative incidence of psychiatric disorders

**Fig. 1:**
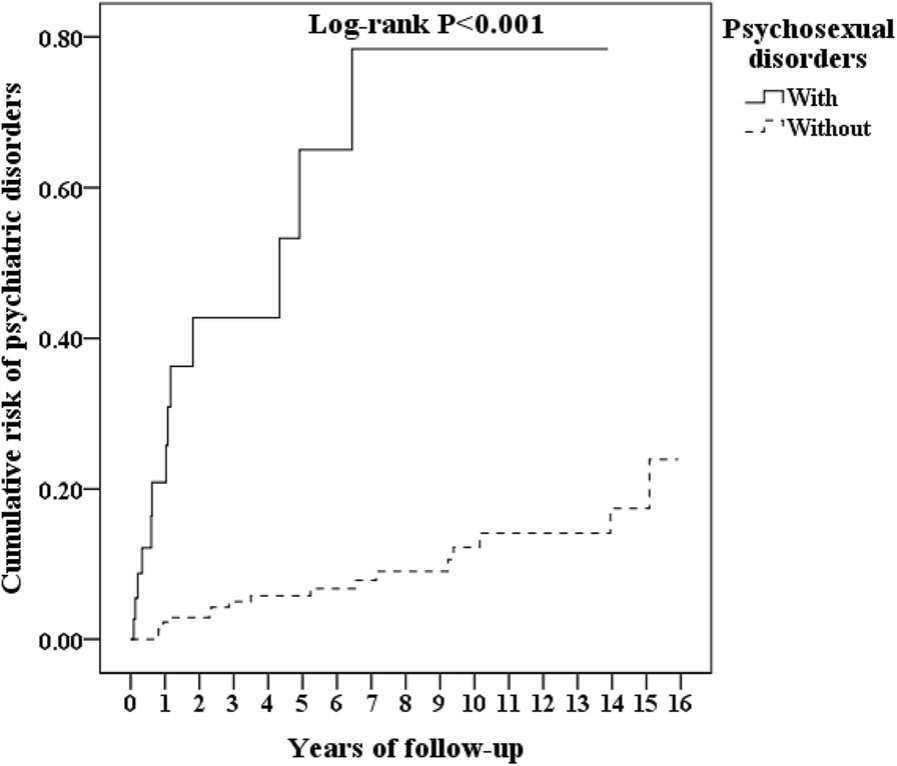
Kaplan-Meier for the cumulative incidence of psychiatric disorders aged 20 and over stratified by psychosexual disorders with the log-rank test

There were 98 in the cohort with psychosexual disorders and 119 in the comparison cohort that developed psychiatric disorders (3444.66 vs 736.07 per 100,000 person-year). Figure 1 depicts that the difference was statistically significant in the Kaplan-Meier survival analysis (log-rank, *p* < 0.001).

### Changes of psychosexual disorders in the follow-up period, 2000–2015

**Fig. 2:**
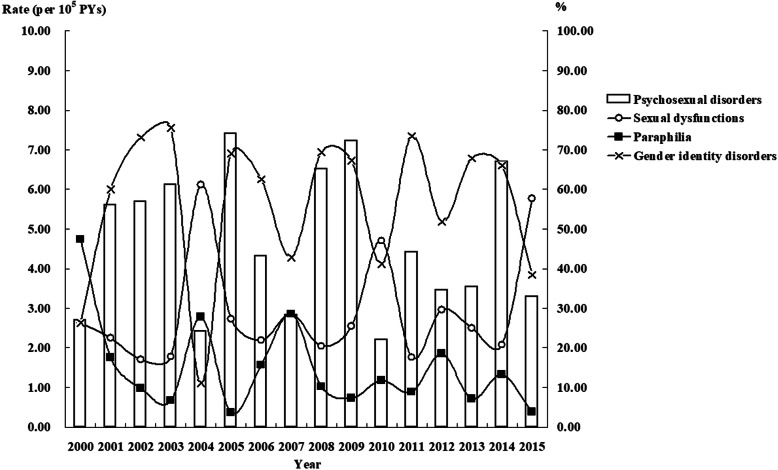
Rate and subgroup proportions of psychosexual disorders in the study period

Figure 2 reveals that there was no significant difference between the beginning and the end-point of the follow-up in all these psychosexual disorders, between 2000 and 2015. Besides, the treatment prevalence of the female psychosexual disorders was 0.007% of the sexual dysfunctions, paraphilias were around 0.004%, and the female-to-male (FTM) gender identity disorder was 0.017%, during the 15-year follow-up.

### HR analysis of psychiatric disorders in patients with psychosexual disorders

The multivariate Cox regression model showed that the adjusted HR of the psychosexual disorders cohort in the development of psychiatric disorders was 9.848 (95% CI = 7.298—13.291, *p* < 0.001), after adjustment for age, marital status, education, comorbidity (CCI scores), urbanizations/areas of residence, insurance premiums, seasons of visits, and levels of medical facilities, as compared to the control group (Table 2).

**Table 2:** Factors of psychiatric disorders stratified by variables listed in the table by using the Cox regression model

Psychosexual disorders (With vs. Without)	With			Without						
Stratified	Event	PYs	Rate (per 10^**5**^ PYs)	Event	PYs	Rate (per 10^**5**^ PYs)	Adjusted HR	95% CI	95% CI	***P***
**Total**	98	2844.99	3444.66	119	16,167.03	736.07	9.848	7.298	13.291	< 0.001
**Age group (years)**										
20–49	70	1823.05	3839.71	91	9797.14	928.84	8.699	6.447	11.740	< 0.001
≧50	28	1021.93	2739.91	28	6369.89	439.57	13.117	9.720	17.703	< 0.001
**Married**										
Yes	38	1400.98	2712.38	48	7754.07	619.03	9.221	6.833	12.444	< 0.001
No	60	1444.00	4155.12	71	8412.96	843.94	10.361	7.678	13.983	< 0.001
**Education (years)**										
< 12	27	1034.02	2611.17	20	8026.18	249.18	22.051	16.341	29.761	< 0.001
≧12	71	1810.97	3920.56	99	8140.85	1216.09	6.784	5.028	9.156	< 0.001
**Insured premium (NT$)**										
< 18,000	67	1512.44	4429.93	73	7558.18	965.84	9.652	7.153	13.026	< 0.001
18,000-34,999	24	851.67	2818.01	38	4311.00	881.47	6.728	4.986	9.080	< 0.001
≧35,000	7	480.88	1455.66	8	4297.85	186.14	16.457	12.195	22.210	< 0.001
**Season**										
Spring	21	535.28	3923.14	28	3746.20	747.42	11.046	8.185	14.907	< 0.001
Summer	28	1081.07	2590.02	35	4793.88	730.10	7.465	5.532	10.075	< 0.001
Autumn	21	205.32	10,228.13	28	3561.62	786.16	27.378	20.289	36.950	< 0.001
Winter	28	1023.31	2736.22	28	4065.33	688.75	8.360	6.195	11.283	< 0.001
**Urbanization level**										
1 (The highest)	42	1173.26	3579.77	42	5526.97	759.91	9.913	7.346	13.379	< 0.001
2	35	1081.95	3234.89	42	5927.50	708.56	9.607	7.120	12.966	< 0.001
3	0	267.33	0.00	21	1360.70	1543.33	0.000	–	–	0.781
4 (The lowest)	21	322.44	6512.77	14	3351.86	417.68	32.813	24.316	44.285	< 0.001
**Level of care**										
Medical center	21	932.95	2250.93	21	5334.73	393.65	12.033	8.917	16.240	< 0.001
Regional hospital	42	1076.64	3901.02	77	6635.84	1160.37	7.075	5.243	9.548	< 0.001
Local hospital	35	835.39	4189.64	21	4196.46	500.42	17.618	13.056	23.778	< 0.001

*PYs* Person-years, *Adjusted HR* Adjusted Hazard ratio: Adjusted for the variables listed in Table 1, *CI* Confidence interval

### Types of psychiatric disorders in female patients with psychosexual disorders

Table 3 depicts that the cohort with psychosexual disorders, including sexual dysfunctions, paraphilias, and gender identity disorders, were associated with the risk of psychiatric disorders.

**Table 3:** Factors of psychiatric disorders stratified by psychosexual disorders subgroup by using Cox regression model

Psychosexual disorders	Adjusted HR	95% CI	95% CI	***P***
**Overall (*N*** **= 98)**	9.848	7.298	13.291	< 0.001
**Sexual dysfunctions (*N*** **= 42)**	6.488	4.808	8.757	< 0.001
**Paraphilias (*N*** **= 21)**	33.366	24.726	45.031	< 0.001
**Gender identity disorders (*N*** **= 35)**	12.286	9.105	16.581	< 0.001

*PYs* Person-years, *Adjusted HR* Adjusted Hazard ratio: Adjusted for the variables listed in Table 1, *CI* Confidence interval

Also, there were no significant differences in the times of the psychiatric visits between the two cohorts, even though the cohort with psychosexual disorders had more psychiatric visits than the comparison cohort (3.82 [standard deviation (SD) ± 4.06] vs 3.15 [SD ± 3.97]), without a statistical difference (*p = 0.001*) (Table S2).

## Discussion

### Association between psychosexual disorders and the risk of psychiatric disorders

The adjusted HR was 9.848 (95% CI = 7.298—13.291, *p* < 0.001) in the association between the psychosexual disorders and psychiatric disorders, and the female patients with psychosexual disorders had a 9.8-fold increase in the risk of psychiatric disorders, after the adjustment of age, monthly income, urbanization level, geographic region, and comorbidities. The Kaplan-Meier analysis demonstrated that the cohort with psychosexual disorders had a significantly higher 15-year psychiatric disorders cumulative incidence than the comparison cohort. To the best of our knowledge, this is the first study on the topic of an association between female patients with psychosexual disorders and the risk of psychiatric morbidity. This finding could serve as a reminder for the clinicians to pay much more attention to these patients because of the issues about psychiatric disorders.

### Comparison of this study to previous literature

Previous studies have shown the association between psychosexual disorders and psychiatric disorders that included antidepressant-related sexual dysfunctions in patients with depressive or anxiety disorders [16, 45–47], female paraphilia focused and the personality disorders on the forensic psychiatric topics [14, 15], and the FTM gender disorders and depression, post-traumatic stress disorder, anxiety disorders and suicides [12, 13, 48]. However, these studies were mostly conducted in cross-section methods, and our study is unique for the retrospective cohort design, from a larger population-based database. Besides, male patients with psychosexual disorders have been associated with an increased risk of anxiety disorders, depressive disorders, bipolar disorders, sleep disorders, and psychotic disorders, respectively [33]. There were several differences in the risk of different psychiatric disorders in these two studies. The underlying reasons for the difference of risk for psychiatric disorders, between female patients with psychosexual disorders, needs further studies.

### Treatment prevalence of psychosexual disorders in this study

Previous studies revealed that the prevalence of female sexual dysfunctions was 30—60%, in different countries [49–52], but we found that there was 0.007% of sexual dysfunctions in this sample of 15-year of follow-up. In the present study, there were 70 paraphilia patients from the database, and the treatment prevalence of female paraphilias was around 0.004% in this LHID. The prevalence of the female paraphilias were 2% in exhibitionistic behaviors in previous studies [25, 53], 4% in voyeuristic behaviors [25, 53], 0.4% in transvestic fetishism [54], and 1% in sadomasochistic activity [55], from surveys in the population of Sweden [25, 53], and Australia [55]. Previous reports have shown that there were 0.003% in Belgium, [56], 0.82% in Japan [57], and 0.023–0.058% in the United States veteran’s populations [12, 58] of FTM gender identity disorder. Furthermore, the present study found that the treatment prevalence of FTM gender identity disorder, was 0.017%, in the duration of the 15 years of follow-up. The discrepancy of the prevalence might be the difference of studies from a claims database or the survey. Cultural differences might also contribute to this difference: previous studies have shown that females have more difficulties in their help-seeking for sex-related problems in Asian countries [59, 60]. However, the present study is the first one for females with psychosexual disorders and the risk of psychiatric disorders in an Asian country.

### Possible mechanisms for the increased risk of psychiatric disorders in patients with psychosexual disorders

In the present study, female patients with sexual dysfunctions were associated with psychiatric disorders. There are several neurodevelopmental, endocrine, and psychological factors related to the linkage between these two groups of disorders. The stress from the suffering of sexual dysfunction [61, 62], paraphilias [63, 64], and gender identity disorders [65–67], might well contribute to the association between these psychosexual disorders and the risk of psychiatric disorders, such as anxiety, depressive, or sleep disorders. One study has found that hyperprolactinemia seems to play a role in the pathogenesis of hypoactive sexual desire disorder, one of the female sexual dysfunctions [68], and hyperprolactinemia might induced psychiatric disorders, such as depression and anxiety [69–72].

Evidence suggests that female and male brains are different in the mean volumes of the hippocampus, amygdala, and thalamus [73], the concentration of estrogen or androgen receptors [74], and the total brain, cerebrum, and cerebellum volumes [75]. Thus, the difference in the brain anatomy and neuronal signaling pathways are more closely aligned with a person’s perceived gender identity, and individuals with discordant gonadal and brain developments might experience psychological challenges for the generalized dissatisfaction with their biological sex [76]. Besides, paraphilias and depression might share a common dysregulation of this monoaminergic pathway in these patients [11, 77].

Psychological, social, and cultural factors might also contribute to both psychosexual disorders and psychiatric disorders. Previous studies have shown that patients with paraphilias might suffer emotional distress, social embarrassment [4], and stigma [5]. For example, a study from Turkey has found that patients with vaginismus have higher levels of depression and anxiety [78]. Phobic defense mechanisms [79], the rejection of the female role, and religious orthodoxy which regards sex as dirty or shameful [80] are the psychosocial factors that contribute to vaginismus, depression, and anxiety [78].

### Limitations

The present study has several limitations that warrant consideration. First, similar to previous studies using the NHIRD on psychosexual disorders [32, 81–83], we were unable to evaluate the severity, weakness severity, laboratory parameters, or psychological assessments in the patients with psychosexual disorders, since the data were not recorded in the NHIRD. Second, the genetic, psychosocial, and environmental factors, were not included in the dataset. Third, even though we have excluded the patients diagnosed with psychiatric disorders before 2000, or before their first visit for any psychosexual disorders, there is the possibility of the protopathic bias, in which some patients could have been introduced into this study by subjects who have an undiagnosed disease. Fourth, although paraphilias and gender dysphoria are distinct categories, there is some evidence for an overlap between paraphilias and gender dysphoria [84]. The combination of distinct entities, in a single heterogeneous category of psychosexual disorders, is a limitation when discussing the results of the data analysis. Fifth, there is a possibility that the high prevalence of psychiatric disorders, among female patients with psychosexual disorders, is due to the high utilization of psychiatric services. However, as shown in Table S2, there were no significant differences in the times of psychiatric visits between the two cohorts.

## Conclusion

Female patients who suffer from psychosexual disorders have a 9.8-fold increase in the risk of psychiatric disorders, and this finding should serve as a timely reminder for the clinicians to pay much more attention to these patients because of their mental health issues.

## Supplementary Information

**Additional file 1: Table S1.** ICD-9-CM codes of Psychosexual disorders.

**Additional file 2: Table S2.** Frequency of psychiatric service.

## Data Availability

Data are available from the National Health Insurance Research Database (NHIRD) published by the Taiwan National Health Insurance (NHI) Administration. Due to legal restrictions imposed by the government of Taiwan concerning the “Personal Information Protection Act”, data cannot be made publicly available. Requests for data can be sent as a formal proposal to the NHIRD (https://dep.mohw.gov.tw/DOS/lp-2506-113.html).

## Abbreviations

CCI: Charlson Comorbidity Index
CI: Confidence interval
FTM: Female-to-male
HR: Hazard ratio
ICD-9-CM: International Classification of Diseases, 9th Revision, Clinical Modification
LHID: Longitudinal Health Insurance Database
NHIRD: National Health Insurance Research Database
NT$: New Taiwan Dollars
