# Supplementary material for: Psychiatric disorders in female psychosexual disorders—a nationwide, cohort study in Taiwan: Psychiatric disorders and female psychosexual disorders
Source: BMC Psychiatry. 2021 Jan 28;21:63. doi: 10.1186/s12888-021-03060-1 (PMC7845000; doi:10.1186/s12888-021-03060-1)
Supplement: Supplementary file 1 — Additional file 1: Table S1. ICD-9-CM codes of Psychosexual disorders. [file 12888_2021_3060_MOESM1_ESM.docx]

**Table S1. ICD-9-CM codes of Psychosexual disorders**

| **PSD/Psychiatric disorders** | **ICD-9-CM codes** | |
| --- | --- | --- |
| **Sexual dysfunctions** |  | 302.7, 607.84 |
| **Gender identity disorders** |  | 302.5, 302.6 |
| **Paraphilia** | **Exhibitionism** | 302.4 |
|  | **Fetishism** | 302.81 |
|  | **Frotteurism** | 302.89 |
|  | **Pedophilia** | 302.2 |
|  | **Sexual masochism** | 302.83 |
|  | **Sexual sadism** | 302.84 |
|  | **Transvestic fetishism** | 302.3 |
|  | **Voyeurism** | 302.82 |
|  | **Other paraphilia** | 302.85 |
|  | **Paraphilia, not otherwise specified** | 302.9 |
| **Dementia** |  | 290.0, 290.1x, 290.2x, 290.3, 290.4x, 290.8, 290.9, and 331.0 |
| **Anxiety disorders** |  | 300 |
| **Depressive disorders** |  | 296.2, 296.3, 300.4, and 311 |
| **Bipolar disorders** |  | 296.0, 296.4-296.8 |
| **Eating disorders** |  |  |
|  | **Anorexia nervosa** | 307.1 |
|  | **Bulimia nervosa** | 307.51 |
|  | **Other eating disorders** | 307.59 |
|  | **Eating disorders, not otherwise specified** | 307.5, except 307.51, 307.59 |
| **Sleep disorders** |  | 307.4, 780.5 |
| **Psychotic disorders** |  | 295, 297-298 |

**PSD: psychosexual disorders; ICD-9-CM: International Classification of Diseases, Ninth Revision, Clinical Modification**
