# Supplementary material for: Psychiatric disorders in female psychosexual disorders—a nationwide, cohort study in Taiwan: Psychiatric disorders and female psychosexual disorders
Source: BMC Psychiatry. 2021 Jan 28;21:63. doi: 10.1186/s12888-021-03060-1 (PMC7845000; doi:10.1186/s12888-021-03060-1)
Supplement: Supplementary file 2 — Additional file 2: Table S2. Frequency of psychiatric service. [file 12888_2021_3060_MOESM2_ESM.docx]

**Table S2. Frequency of psychiatric service**

|  | **Overall**  (n = 2,240) | **The cohort with psychosexual disorders**  (n = 560) | **The cohort without psychosexual disorders**  (n = 1,680) | ***P*** |
| --- | --- | --- | --- | --- |
| **At the baseline** | 3.32 ± 4.00 | 3.82 ± 4.06 | 3.15 ± 3.97 | 0.001 |
| **At the endpoint** | 3.87 ± 4.28 | 4.18 ± 4.49 | 3.77 ± 4.21 | 0.050 |

| ***P:* t-test** |
| --- |
